# Development of Composite PCMs by Incorporation of Paraffin into Various Building Materials

**DOI:** 10.3390/ma8020499

**Published:** 2015-02-05

**Authors:** Shazim Ali Memon, Wenyu Liao, Shuqing Yang, Hongzhi Cui, Syed Farasat Ali Shah

**Affiliations:** 1 Guangdong Provincial Key Laboratory of Durability for Marine Civil Engineering, College of Civil Engineering, Shenzhen University, Shenzhen 518060, China; E-Mails: shazimalimemon@gmail.com (S.M.); liaowenyu@email.szu.edu.cn (W.L.); 2 Department of Civil Engineering, COMSATS Institute of Information Technology, Abbottabad Campus, Abbottabad 22010, Pakistan; E-Mail: farasatali@ciit.net.pk; 3 Department of Civil and Environmental Engineering, School of Engineering, the Hong Kong University of Science and Technology, Hong Kong 999077, China; E-Mail: syangaq@connect.ust.hk

**Keywords:** building materials, latent-heat storage, composite phase-change material (CPCM), phase change materials, paraffin, Kaolin, ground granulated blast-furnace slag (GGBS)

## Abstract

In this research, we focused on the development of composite phase-change materials (CPCMs) by incorporation of a paraffin through vacuum impregnation in widely used building materials (Kaolin and ground granulated blast-furnace slag (GGBS)). The composite PCMs were characterized using environmental scanning electron microscopy (ESEM), Fourier transform infrared spectroscopy (FT-IR), differential scanning calorimetry (DSC) and thermogravimetric analysis (TGA) techniques. Moreover, thermal performance of cement paste composite PCM panels was evaluated using a self-designed heating system. Test results showed that the maximum percentage of paraffin retained by Kaolin and GGBS was found to be 18% and 9%, respectively. FT-IR results show that CPCMs are chemically compatible. The phase-change temperatures of CPCMs were in the human comfort zone, and they possessed considerable latent-heat storage capacity. TGA results showed that CPCMs are thermally stable, and they did not show any sign of degradation below 150 °C. From thermal cycling tests, it was revealed that the CPCMs are thermally reliable. Thermal performance tests showed that in comparison to the control room model, the room models prepared with CPCMs reduced both the temperature fluctuations and maximum indoor center temperature. Therefore, the prepared CPCMs have some potential in reducing peak loads in buildings when applied to building facade.

## 1. Introduction

With the growth in the global population and the increased use of natural resources by humankind, environmental problems continue to arise. More focus should be placed on the use of renewable energy sources that reduce environmental pollution and, at the same time, improve human quality of life. Among renewable energy sources, solar energy can be directly developed and used without mining and transport.

Phase change materials (PCMs) are “latent” thermal storage materials possessing a large amount of heat energy stored during phase change. PCMs latent-heat storage can be achieved through solid–solid, solid–liquid, solid–gas and liquid–gas phase change [1]. A solid–liquid PCM works on the principle that as the temperature increases, the material stores energy by changing from a solid to a liquid at a discrete temperature. Similarly, when the temperature decreases, the PCM releases heat by changing from a liquid to a solid. Among the studied solid–liquid PCMs, paraffins have been utilized widely for latent-heat thermal energy-storage applications because of their high latent heat and favorable thermal characteristics, such as little or no supercooling, low vapor pressure, and good thermal and chemical stability.

The combination of building materials and PCMs is an efficient way to increase the thermal energy-storage capacity of building components for the purpose of direct, thermal energy storage in buildings [2,3]. The temperature differences between night and day ensure that the PCM passes through alternating melting and solidification stages as the exterior temperature rises and falls, respectively, thereby functioning as a heating and cooling system for a building. This means that during the daytime, the PCM in building materials absorbs surplus heat by melting. At cooler temperatures during the night, the PCM becomes solid, and the heat is released back into the environment. A similar thermal cycle repeats daily, and solar thermal energy can be utilized effectively by means of PCMs in buildings.

Meshgin and Xi [4] experimentally investigated the utilization of PCM, as sand replacement and additive, in Portland cement concrete. Test results showed that the loss of compressive strength due to the addition of PCM as replacement of sand was not as high when PCM was used as an additive. For up to 20% sand replacement by PCM, the strength loss was not significant for structural applications. Moreover, the specific heat of the concrete increased considerably. The data from flexural test, drying shrinkage test and microstructure analysis provided a good understanding of the PCM-modified concrete. Overall, the results showed that it is quite promising to use PCM in concrete to improve its insulation capacity with decreased thermal conductivity and, at the same time, it is possible to keep the strength loss of the concrete in an acceptable range.

To avoid PCM leakage and utilize the materials simply, composite PCMs (CPCMs) have been developed and have attracted the interest of many researchers, especially in the last decade. However, the following issues still need to be addressed.

(a) Some of the researchers have focused on the utilization of a Eutectic PCM for the development of CPCMs. However, a limited amount of test data is available on their thermo-physical properties [3,5].
(b) Most of the research has not focused on the thermal performance of CPCMs [6,7,8,9,10,11,12,13,14], *i.e.*, scale models are not constructed to determine the temperature variations within a room. In other words, the validity of the reported results is uncertain.

Therefore, in this study, we used a paraffin as a PCM and building materials (*i.e.*, Kaolin and ground granulated blast-furnace slag (GGBS)) as supporting materials for PCM to composite two new kinds of CPCMs. It is worth mentioning here that Kaolin and GGBS are mineral admixtures and have been extensively used in mortar and concrete to improve strength and durability [15,16,17]. Considering these benefits, Kaolin and GGBS are potential candidates for thermal energy storage in buildings. It is believed that the utilization of Kaolin and GGBS for the purpose of thermal energy storage in buildings will open up new opportunities for the use of Kaolin and GGBS.

## 2. Experimental Investigation

In this section are the details related to the development of CPCMs through the incorporation of paraffin into building materials. The details, including the materials used, preparation, and test methods for characterization of CPCMs, are covered.

### 2.1. Materials and Preparation of the CPCMs

**Figure 1:**
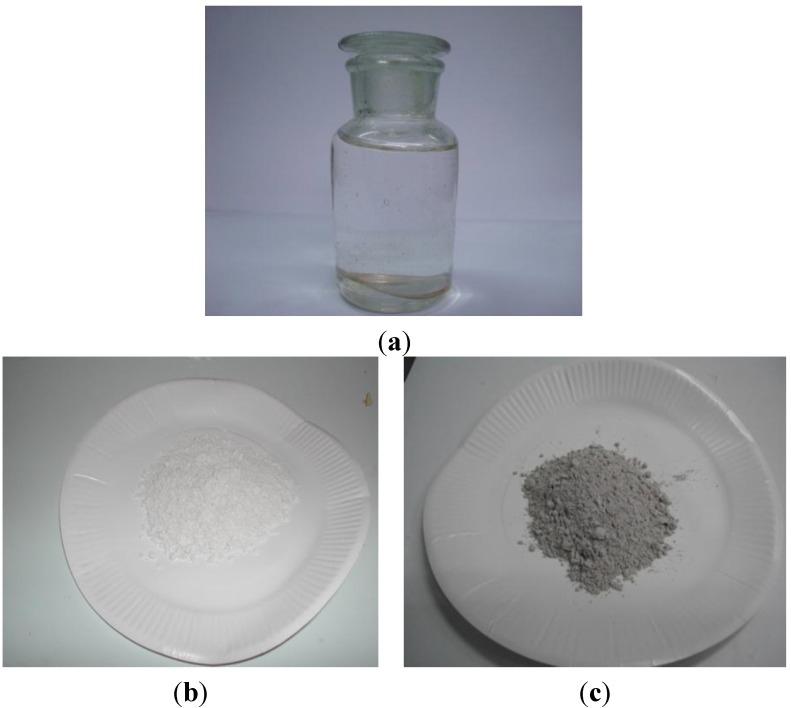
Materials used in this research: (**a**) Paraffin; (**b**) Kaolin; (**c**) ground granulated blast-furnace slag (GGBS).

A technical-grade paraffin made by China Petroleum & Chemical Corporation (Nanyang, China) was used as the PCM, while Kaolin and GGBS were used as container for the PCM (Figure 1). These building materials were sieved through a 150 µm sieve and dried at 105 °C for 24 h before use. The chemical composition of these building materials is given in Table 1. The CPCMs were prepared using vacuum impregnation. The following procedure was adopted. First, the supporting materials (Kaolin and GGBS) with different mass fractions were put inside a vacuum chamber, where it was vacuumed for 90 min at a vacuum pressure of 65 kPa. The melted PCM was then allowed to flow and cover the maximum surface area of the sample. Subsequently, the vacuum pump was switched off, and air was allowed to enter for 30 min to force the PCM to penetrate into the pores of the supporting material. The composite PCM was then placed in an air-conditioned laboratory below the melting point of PCM for 24 h to ensure solidification of the PCM. The optimum percentage of PCM retained by the supporting material was determined by performing leakage test and was designated the composite PCM (CPCM). In this test, the composite PCM was placed in an oven above the melting point of PCM for 30 min so as to observe PCM leakage in the melted state.

**Table 1:** Chemical composition of building materials.

Chemical Composition	Kaolin (%)	GGBS (%)
Silicon dioxide (SiO_2_)	46	31.7
Aluminum oxide (Al_2_O_3_)	38	14.5
Ferric oxide (Fe_2_O_3_)	0.73	1.37
Titanium dioxide (TiO_2_)	0.19	-
Calcium oxide (CaO)	0.02	38.5
Magnesium oxide (MgO)	0.06	8.13
Sodium oxide (Na_2_O)	0.03	-
Potassium oxide (K_2_O)	0.65	-
Sulfate as SO_3_	-	2.61
Loss on ignition	13.7	-
Others		3.19

### 2.2. Test Methods for Characterization of the CPCMs

#### 2.2.1. Environmental Scanning Electron Microscopy (ESEM)

The microscopic morphology of Kaolin and GGBS and the prepared CPCMs were observed using a XL30 FEG ESEM (FEI, Hillsboro, OR, USA) operated in secondary-electron detection mode under low vacuum and at an accelerating voltage of 15 kV. The powdered sample was placed on carbon tape attached to a sample holder. Several regions were observed at different magnifications to ensure that the observed structures were representative of the sample.

#### 2.2.2. Chemical Compatibility of the CPCMs

The chemical compatibility between the components of the CPCMs was evaluated using FT-IR spectroscopy (Perkin-Elmer, Waltham, MA, USA). The spectra were recorded on a Perkin-Elmer spectrometer, model No. PE-100 (Perkin-Elmer). The procedure for obtaining the FT-IR spectra is as follows.

The sample and the KBr were heated for 24 h at 105 °C. They were then mixed in 1:20–1:30 (powder: KBr) ratios in a controlled-humidity environment. Thereafter, the mixed sample was pressed at 10 ton for 1 min in “The Specac 15 ton Manual Hydraulic Presses”. Finally, the obtained KBr pellet was placed in the sample container, and an infrared spectrum was obtained using the Spectrum Version 5.0.1 software (Perkin-Elmer). The scanning parameters were: spectral range of 4000–400 cm^−1^, 16 scans and resolution of 4 cm^−1^. In order to remove the instrumental characteristics from the spectrum, a background spectrum was also measured. This measurement was performed by taking the reading with no sample in the sample compartment.

#### 2.2.3. Thermal Properties of the CPCMs

The thermal properties, *i.e.*, the phase-change temperature and thermal energy stored per unit mass of the composite PCMs were determined using a TA instrument, model MDSC2910 (TA Instruments, New Castle, DE, USA), while the thermal properties were extracted using the TA software, Universal Analysis 2000 [18]. The phase change temperature is divided into starting, peak and ending temperatures. The starting and ending temperatures are the temperatures at the intersection of extrapolated baseline and the tangents to the differential scanning calorimetry (DSC) (NETZSCH, Selb, Germany) curve drawn at the inflection points to the left and right side of the peak while the peak temperature is the temperature at the peak point of DSC curve. The thermal heat stored in the unit weight of PCM is obtained by dividing the integrated area between the base line and the DSC curve with temperature rising rate in the DSC test. This value is calculated automatically by the software [19]. Throughout the experiment, nitrogen was used as purge gas at a flow rate of 50 mL·min^−1^. The maximum deviation in the phase-change temperature and latent-heat values were found to be ±0.12 °C and ±0.24 J/g, respectively. The following method log was used.

(a) Equilibrate at 0.00 °C
(b) Isothermal for 3.00 min
(c) Ramp 5.00 °C/min to 40.00 °C
(d) Isothermal for 2.00 min
(e) Ramp 5.00 °C/min to 0.00 °C
(f) End of method

Kheradmand *et al.* [20] studied the effect of the heating/cooling rates (0.1 °C/min, 1 °C/min, 2 °C/min, 4 °C/min, 6 °C/min) on the test results of DSC experiment. It was found that although the overall differences in the thermograms for each rate heating/cooling are smaller, as the rate decreases, the accumulated specific enthalpy is almost constant regardless of the heating/cooling rate. Since in this research we have paid more attention on the specific enthalpy, the temperature variation rate of 5 °C/min has been adopted so as to not influence the discussion.

#### 2.2.4. Thermal Stability of the CPCMs

The thermal stability of the CPCMs was determined using a Perkin-Elmer Simultaneous Thermal Analyzer (STA 6000, Perkin-Elmer), while TGA results were extracted using the Pyris software (Perkin-Elmer). Throughout the experiment, nitrogen was used as purge gas at a flow rate of 20 mL·min^−1^.

#### 2.2.5. Thermal Reliability of the CPCMs

The thermal reliability of the CPCMs was evaluated with respect to change in the phase-change temperature and latent heat after a large number of thermal cycling. The thermal cycling consisted of exposing the CPCMs to 30 cycles. This cycling was accomplished by keeping the sample in a controlled laboratory environment for one month (one cycle/day for one month). The details of the temperature regime used for a single cycle are given below.

(a) 1–8 h: the temperature was maintained at 26 °C.
(b) 8–12 h: the temperature was decreased to 18 °C at a rate of 2 °C/h.
(c) 12–20 h: the temperature was maintained at 18 °C.
(d) 20–24 h: the temperature was increased to 26 °C at a rate of 2 °C/h.

After thermal cycling, FT-IR and DSC analyses were repeated to verify the chemical and thermal stability of the CPCMs.

#### 2.2.6. Thermal Performance of Cement-Paste Panels

**Figure 2:**
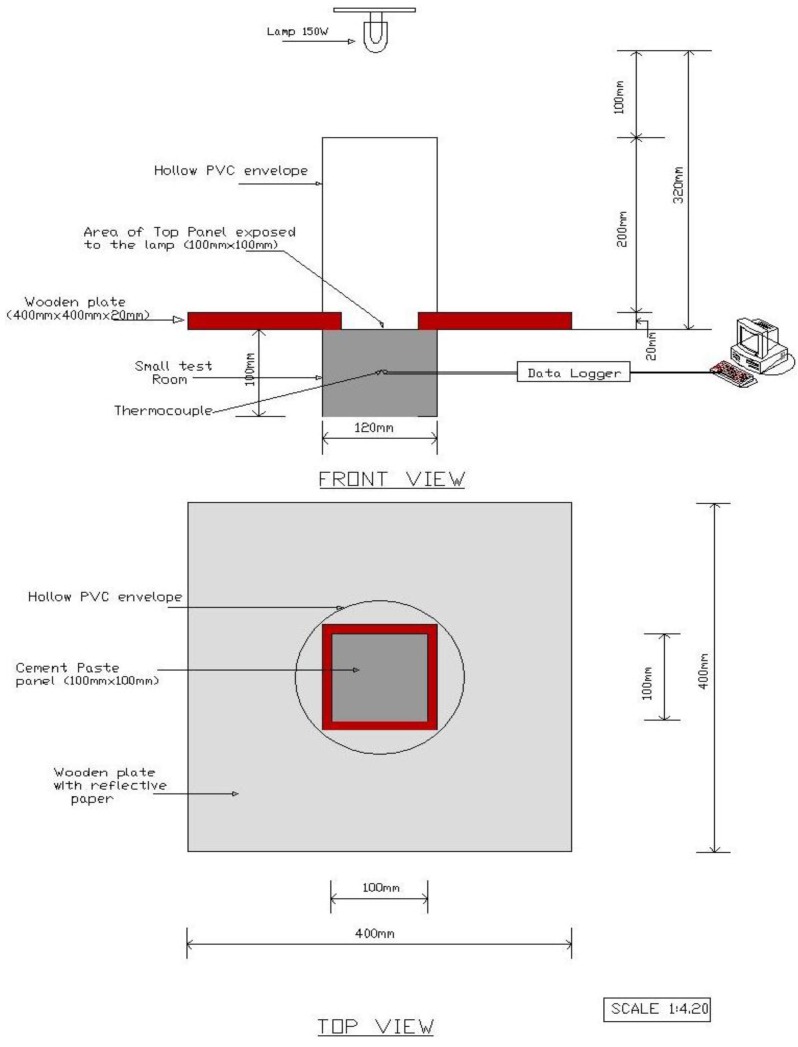
Schematic of thermal performance test.

The thermal performance of cement-paste panels (with and without CPCMs) with dimensions of 100 mm × 100 mm × 10 mm were evaluated at the age of 2 days using a self-designed heating system (Figure 2). The setup consisted of small test room, a 150 W infrared lamp (used as a heating source) placed over the top panel at a distance of 320 mm, a hollow PVC (polyvinyl chloride) envelope coated on the inside with reflective paper to create a uniform and steady temperature field, a wooden plate 400 mm × 400 mm × 20 mm with an opening of 100 mm × 100 mm × 20 mm placed between the PVC envelope and the test room, thermocouple placed in the center of the test room and the computer recording system connected with a data-logger. The purpose of keeping the wooden plate with the top surface coated with reflective paper was to avoid heating the side panels.

The small test room consisted of six panels out of which five were prepared with ordinary cement paste, while the top panel (with and without CPCMs) was used to evaluate the thermal performance. In this test, the top panel was heated for 1 h and was then allowed to cool naturally for another hour. The water-to-binder ratios used for the CPCM panel with GGBS and Kaolin were 0.45 and 0.55, respectively. The different water binder ratios were selected to ensure that the developed CPCM are workable. In addition, in the CPCM panels containing Kaolin and GGBS, 50 wt% of the cement was replaced with CPCM. The physical characteristics of the materials are enlisted in Table 2. It is worth noting that the effect of the enthalpies of the CPCM panels cannot be separated from these measurements (thermal conductivity and specific heat capacity); however, since the difference of the specific heat capacity between the cement paste with and without KO-CPCM/GGBS-CPCM is much smaller than the changes of the enthalpy of the CPCM panels, the thermal performance of the CPCM panels can be ignored in the discussion.

**Table 2:** Physical characteristics of the materials used.

Sample Number	Thermal Conductivity Coefficient (W/mK) (Test temperature 22–30 °C)	Density (kg/m^3^)	Specific Heat Capacity (J/kg·K) (Test Temperature 5 °C/35 °C)	Enthalpy (J/kg)
Cement paste	0.91	2230	1033/1033	/
Cement paste with KO-CPCM	0.77	1980	1180/1228	7842
Cement paste with GGBS-CPCM	0.82	2050	1061/1132	4038

## 3. Test Results and Discussion

### 3.1. Morphology and Optimum Percentage Retained by CPCMs

**Figure 3:**
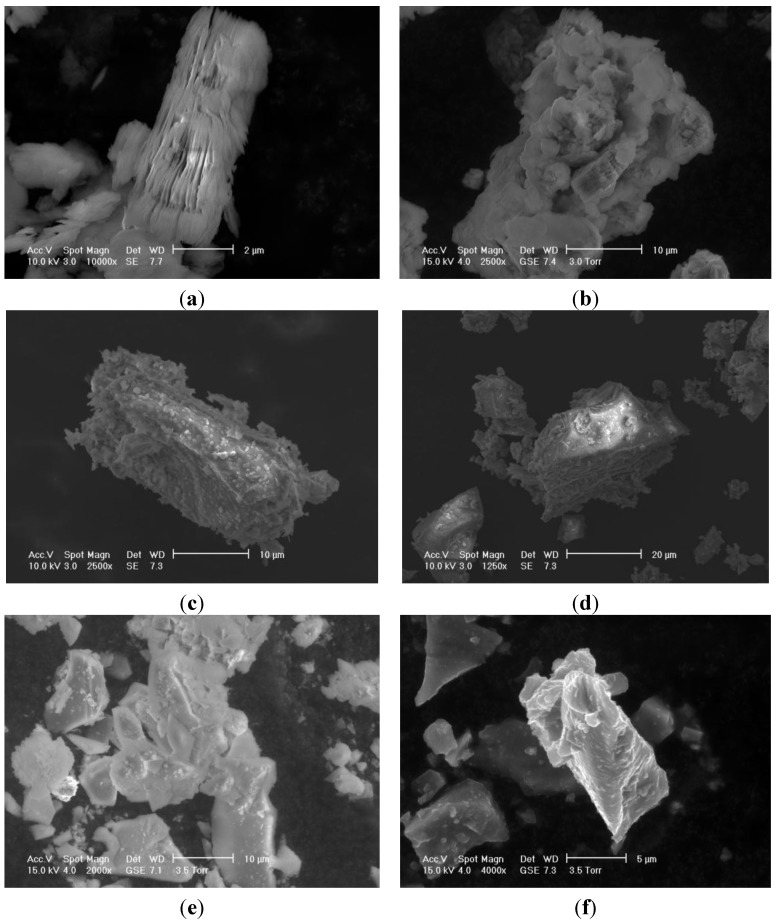
Morphologies in ESEM of (**a**) Kaolin powder; (**b**) Paraffin-Kaolin composite PCM; (**c**,**d**) GGBS particles; (**e**,**f**) Paraffin-GGBS composite PCM.

The morphology of building materials and CPCMs at the microscopic scale is shown in Figure 3. Kaolin particles have a lamellar shape, while GGBS particles show sharp edges and angles with occasionally elongated needled-shaped forms. Paraffin was held by the building materials due to the effect of capillary and surface tension forces, which, in turn, prevented the leakage of the melted PCM. The maximum percentage of paraffin retained by Kaolin and GGBS was found to be 18 and 9 wt%, respectively.

### 3.2. FT-IR Spectroscopy of the CPCMs

**Figure 4:**
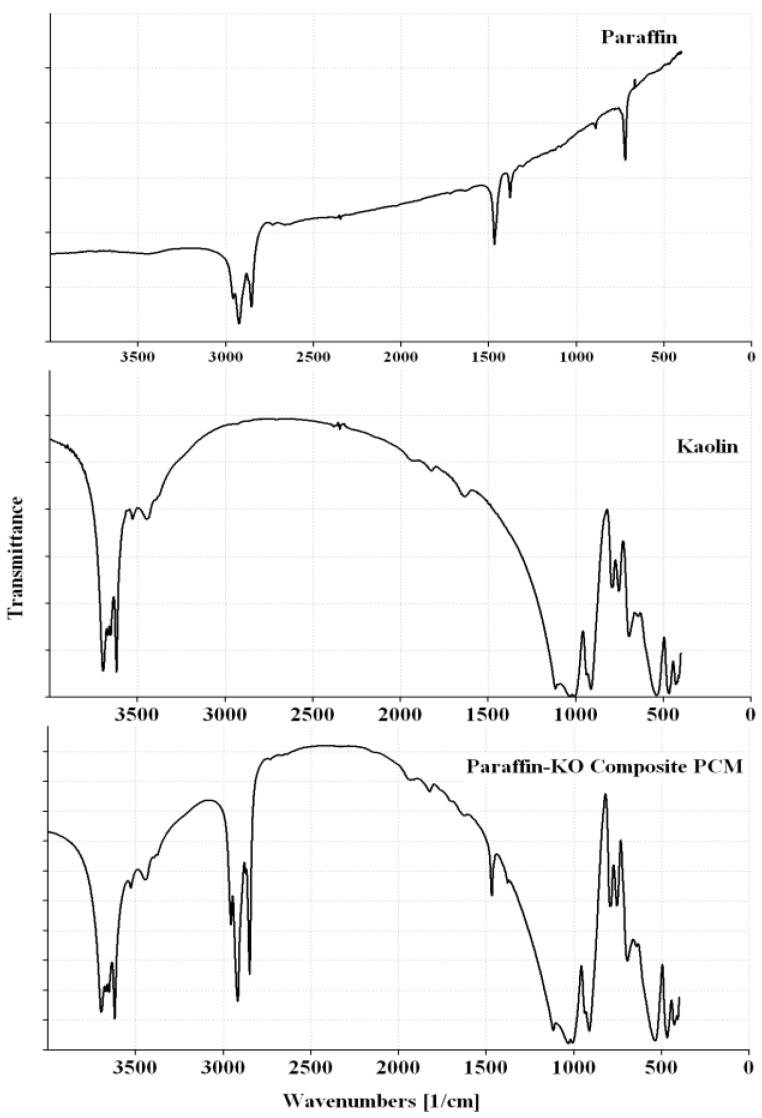
FT-IR spectra of Paraffin, Kaolin and Paraffin-Kaolin composite PCM.

**Figure 5:**
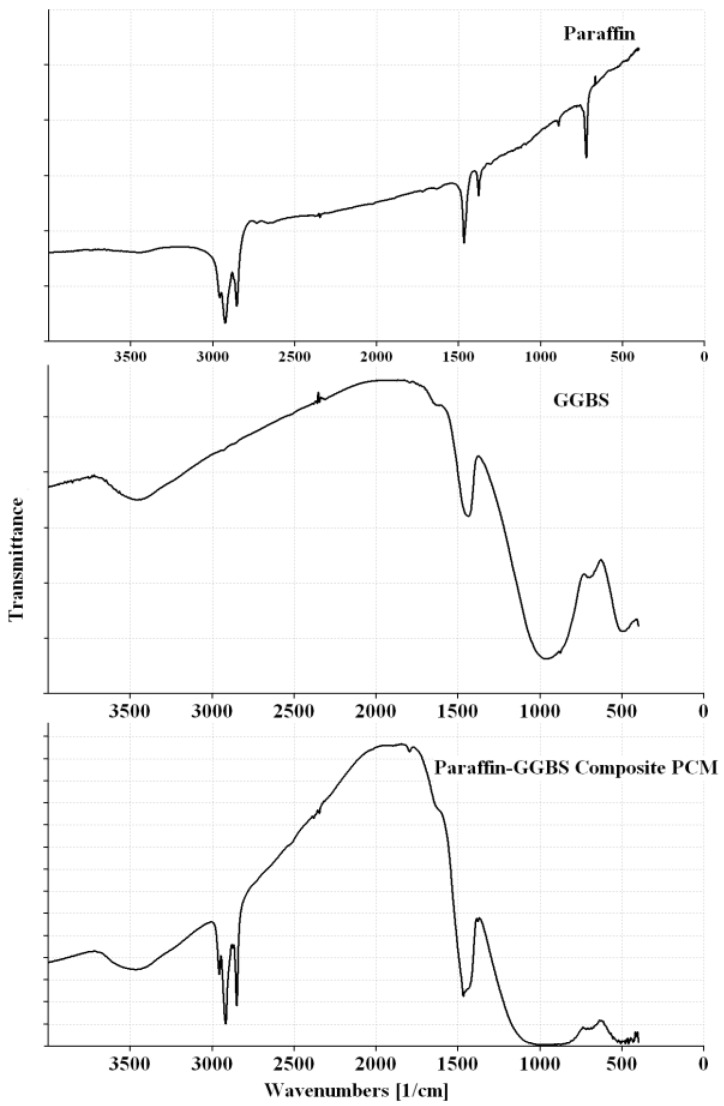
FT-IR spectra of Paraffin, GGBS and Paraffin-GGBS composite PCM.

The FT-IR spectra of the paraffin and CPCMs are shown in Figure 4 and Figure 5, while the significant peaks obtained from the FT-IR spectra along with possible assignments of different bands are shown in Table 3.

**Table 3:** FT-IR absorption bands and assignments.

Band (cm^−1^)	Assignment	Reference
**Paraffin**		
2924	Methylene C–H stretching	[21,22,23]
2853	Methylene C–H stretching	[23,24,25]
1467	Methylene/Methyl C–H bending	[23]
1378	Methyl C–H bending	[23,26,27]
721	Rocking Methylene	[23]
**Kaolin**		
3696, 3669, 3653, 3620	Al–O–H stretching	[28,29,30,31]
3449	H–O–H stretching	[28,29]
1631	H–O–H bending	[28,32,33]
1115, 1031, 1007	Si–O stretching	[28,29,34,35]
**GGBS**		
937, 912	Al–OH bending	[29,32,34]
754	Si–O–Al stretching	[29,34,35]
535	Si–O–Al stretching	[32]
467	Si–O–Si bending	[29,32]
3468	H–O–H stretching	[36,37]
1437	O–C–O stretching	[38,39]
963	Si(Al)–O stretching	[40,41]
711	Si–O–Si (Al) stretching	[41]
494	O–Si–O bending	[40,41]

The FT-IR spectrum of the paraffin (Figure 4) shows peaks at 2924, 2853, 1467, 1378 and 721 cm^−1^. The two characteristic peaks at 2924 and 2853 cm^−1^ are associated with C–H stretching of methylene groups [21,22,23], while the band at 721 cm^−1^ is related to rocking of methylene groups [23,24,25]. The paraffin sample also shows a strong characteristic peak at 1467 cm^−1^ that corresponds to C–H bending of methylene/methyl groups [23] and a weak characteristic peak at 1378 cm^−1^ that is related to C–H bending of methyl groups [23,26,27].

The FT-IR spectra of CPCMs (Figure 4 and Figure 5) show peaks that are already present in Paraffin, Kaolin and GGBS spectra, *i.e.*, no new peak is observed. In the Paraffin-Kaolin composite PCM, the peaks at 3696, 3670, 3652, 3620, 3446, 1116, 1032, 1007, 937, 912, 754, 538 and 467 cm^−1^ are related to Kaolin, while the peaks at 2918, 2850, 1468 and 1378 cm^−1^ correspond to Paraffin. For the Paraffin-GGBS composite PCM, the peaks at 3467, 1444, 958 and 502 cm^−1^ are associated with GGBS, while the peaks at 2919, 2850, 1467, 1378 and 718 cm^−1^ are linked to Paraffin. The CPCMs showed little or no shift in the characteristic peaks clearly depicting that the interactions between the components are physical in nature and prevented the leakage of PCM from the building materials during phase transition of the paraffin [10,19,42,43].

### 3.3. Thermal Properties of the CPCMs

**Figure 6:**
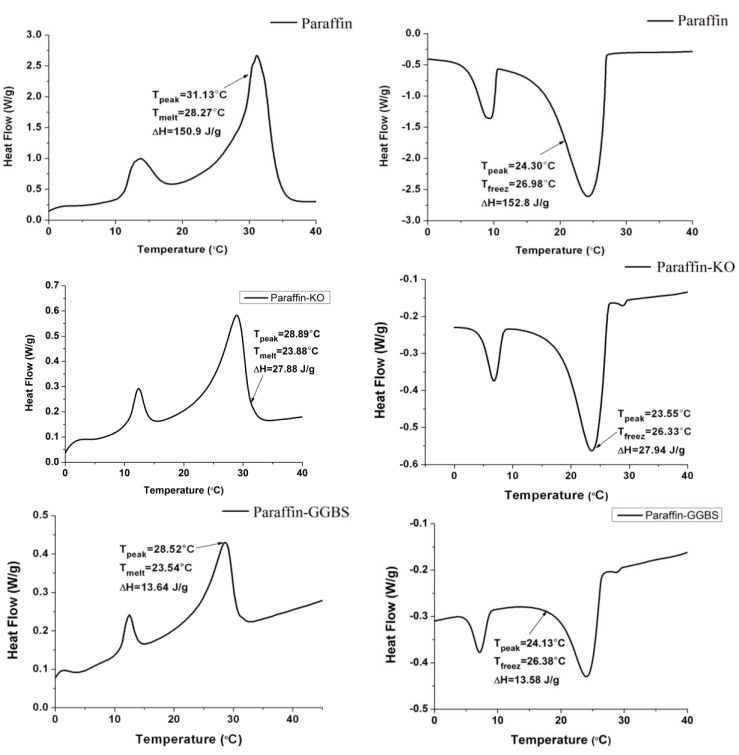
DSC thermograms of the paraffin and CPCMs.

The DSC curves of the paraffin and CPCMs are shown in Figure 6. The melting and freezing temperatures are found to be 28.27 and 26.98 °C for the paraffin, 23.88 and 26.33 °C for Paraffin-Kaolin composite PCM and 23.54 and 26.38 °C for Paraffin-GGBS composite PCM. The decrease in the phase-change temperature is most likely due to the physical interaction between paraffin and building materials as characterized by FT-IR. The results are in agreement with the available literature [19,44,45], in which the researchers found that the interaction between the components of the composite play an important role in deciding the shift direction of the melting point in porous media. Moreover, because the phase change temperature of the composite is close to human comfort temperature, it can be used in building applications as a thermal energy-storage material, especially in the interior walls of buildings, where it can moderate the fluctuations in indoor temperature and improve the indoor thermal environment [14].

The latent heat of melting and freezing stored per unit mass are 150.9 and 152.8 J/g for Paraffin, 27.88 and 27.94 J/g for Paraffin-Kaolin composite PCM and 13.64 and 13.58 J/g for Paraffin-GGBS composite, respectively. The latent-heat storage values of composite PCM are slightly lower/higher than the calculated values, obtained by multiplying the mass content of paraffin in the CPCMs with the latent heat of the paraffin in the pure state. It is worth mentioning here that these materials are already being used in the building industry; therefore, their utilization for energy storage applications would be an additional advantage.

### 3.4. Thermal Stability of the CPCMs

**Figure 7:**
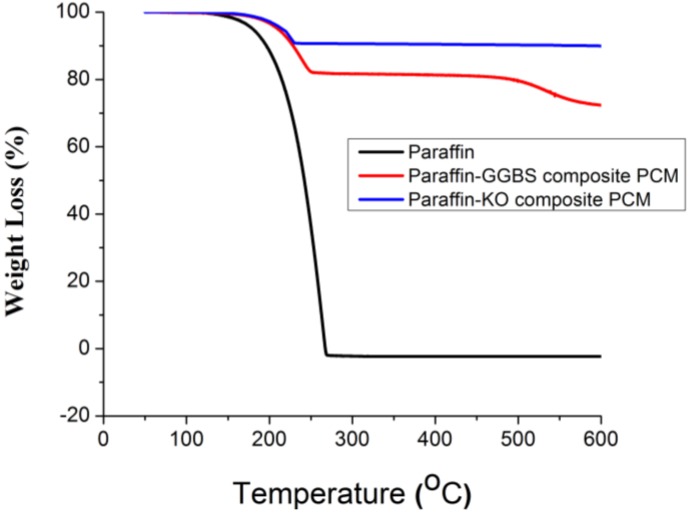
TGA thermograms of the paraffin and CPCMs.

TGA was used to determine the thermal stability of CPCMs. The thermal degradation of the paraffin and CPCMs is shown in Figure 7. The weight-loss process of the paraffin starts at approximately 140 °C and ends at approximately 270 °C with only one step, which corresponds to the volatilization of paraffin. As far as the CPCMs are concerned, weight loss below 150 °C is negligible. Therefore, it can be deduced that the thermal stability of paraffin has increased after being confined in the pores of building materials. It can also be seen that the major weight-loss process for all the CPCMs is completed before that of the pure paraffin. An earlier occurrence of rapid weight loss of CPCMs may be attributed to the difference in physical behavior between free paraffin and pore-confined paraffin. However, the total weight loss of paraffin in Paraffin-Kaolin and Paraffin-GGBS composite PCM measured in the TGA is 17.54% and 8.8%, respectively, which is quite close to the vacuum impregnation results. It demonstrates the homogeneity of CPCMs. As mentioned above, the weight loss in the CPCMs below 150 °C is negligible. Therefore, the prepared composite can be used for thermal energy storage in building applications.

### 3.5. Thermal Reliability of the CPCMs

**Figure 8:**
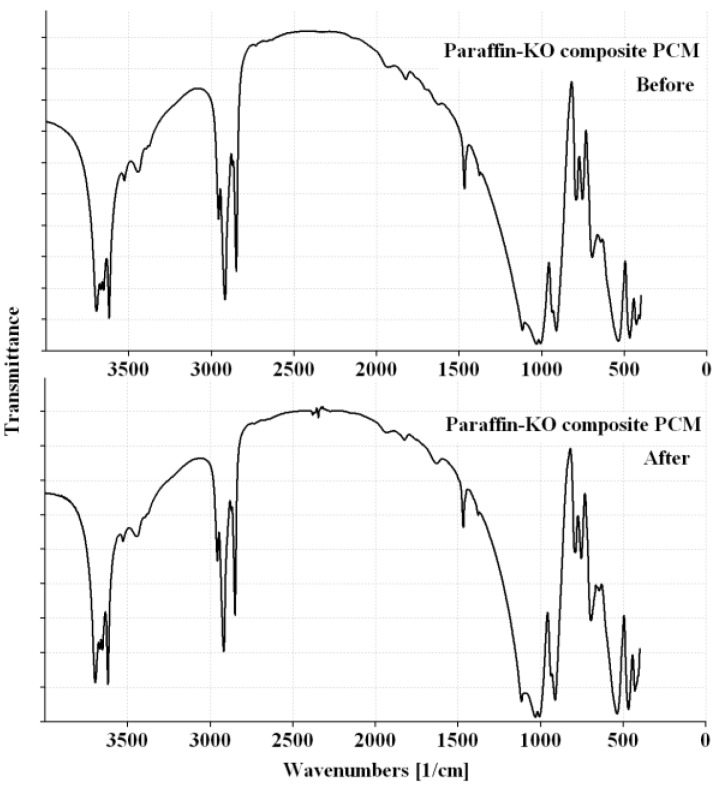
FT-IR spectra of Paraffin-Kaolin composite PCM before and after thermal cycling.

**Figure 9:**
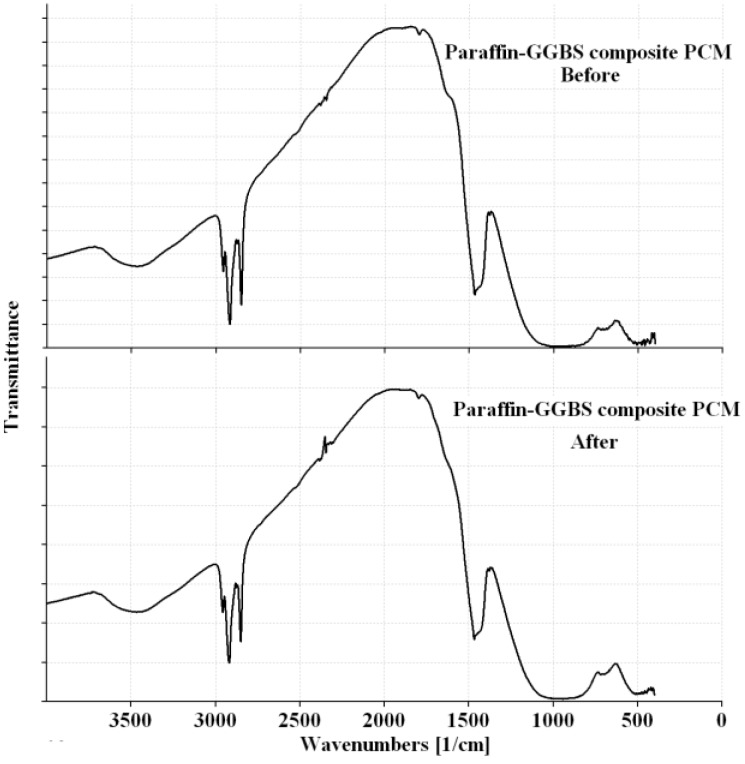
FT-IR spectra of Paraffin-GGBS composite PCM before and after thermal cycling.

**Figure 10:**
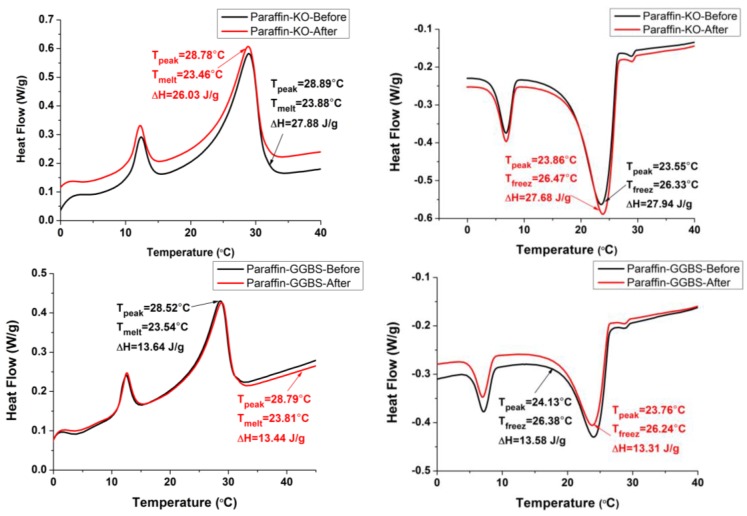
DSC thermograms of CPCMs before and after thermal treatment.

For building applications, the CPCMs should be thermally reliable over a large number of melting and freezing cycles. In other words, the CPCMs should show little or no change in the chemical structure and thermal properties after long-term use [10]. After thermal cycling, the changes in the chemical structure were determined by FT-IR, while the changes in the thermal properties were investigated by DSC. The FT-IR spectra of the composite PCMs before and after thermal treatment are presented in Figure 8 and Figure 9. It can be seen that the shape and peak wavenumber values did not change after thermal cycling. Thus, it can be concluded that the chemical structure of the CPCM was not changed by thermal cycling. The DSC curves of CPCM before and after thermal cycling are shown in Figure 10. The melting and freezing temperatures changed by 0.42 and −0.14 °C for Paraffin-Kaolin composite PCM and −0.27 and 0.14 °C for Paraffin-GGBS composite PCM. The changes observed in the latent-heat storage capacity of melting and freezing were 1.85 and 0.26 J/g for Paraffin-Kaolin composite PCM and 0.2 and 0.27 J/g for Paraffin-GGBS composite PCM. Because the changes observed in the thermal properties are smaller, the developed CPCMs have good thermal reliability and can be used as latent-heat storage materials in buildings.

### 3.6. Thermal Performance of CPCMs

#### 3.6.1. Thermal Performance of Paraffin-Kaolin Composite

**Figure 11:**
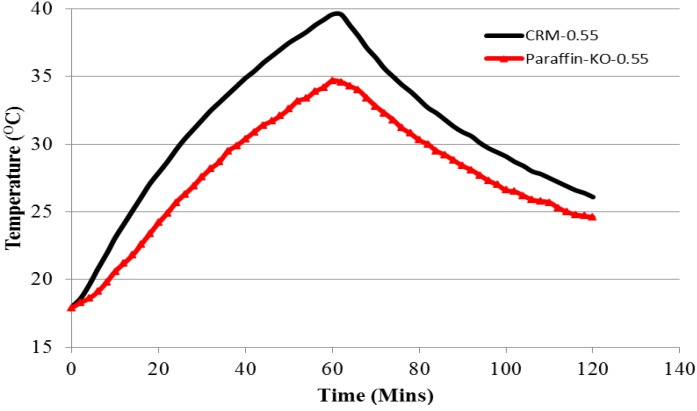
Indoor temperature variation at the center of the room model—Control and Paraffin-Kaolin composite room model.

The thermal performance of the small room model was evaluated by monitoring the indoor temperature variation during a test period of 2 h. The indoor-temperature variation curves of the room model with the top panel prepared with and without Paraffin-Kaolin composite are shown in Figure 11. It can be seen that in comparison to the control room model, the room prepared with PCM-Kaolin composite has a lower indoor temperature during the heating and cooling processes. Additionally, the temperature curves for the room model with Paraffin-Kaolin composite are right-shifted. It is known that the consumption of electricity varies during the day and night according to demand from the building, industrial and commercial sectors. This variation can lead to a differential pricing system for peak and off-peak periods. Thus, Paraffin-Kaolin composite cement-paste panel can be beneficial in such situations by shifting the load away from the peak demand times. Therefore, energy can be purchased at a lower cost during off-peak periods. From the thermal performance test, the maximum temperature values for the control and Paraffin-Kaolin composite room models are 39.6 and 34.7 °C, respectively. This shows that the difference in the maximum indoor center temperature between Control and Paraffin-Kaolin room model is 4.9 °C. Thus, it can be inferred that part of the heating load has been taken by the paraffin in Kaolin. When the cooling curves for the room models are compared, it is found that the indoor temperature decreased by 13.5 and 9 °C for Control and Paraffin-Kaolin room model during the 1 h cooling period. This shows that Paraffin-Kaolin composite room model is helpful in reducing indoor temperature fluctuations. Therefore, it can be concluded that Paraffin-Kaolin composite may be helpful in reducing the energy consumption by decreasing the indoor temperature and hence can be a potential candidate for applications in building facades.

#### 3.6.2. Thermal Performance of Paraffin-GGBS Composite

**Figure 12:**
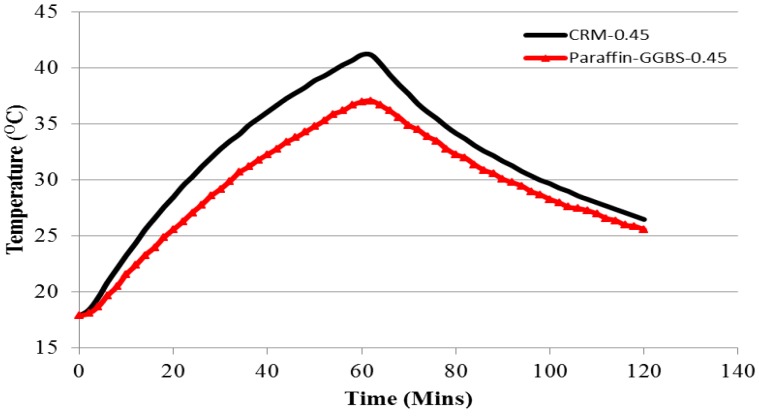
Indoor temperature variation at the center of the room model—Control and Paraffin-GGBS composite room model.

The thermal performance of the control and Paraffin-GGBS composite room model is shown in Figure 12. It can be seen that in comparison to the control room model, the room prepared with Paraffin-GGBS composite exhibits a lower indoor temperature. In addition, the temperature curve of the Paraffin-GGBS composite has a flatter profile and is right-shifted. This response highlights the latent-heat storage performance of the phase-change material during its phase transition from solid to liquid, and consequently the heat transfer by conduction through Paraffin-GGBS composite panel is reduced and retarded. During heating, the Paraffin-GGBS room model exhibited a maximum temperature that was 4.1 °C lower. This result shows that part of the heating load was absorbed by paraffin in GGBS. During cooling for 1 h, the indoor temperature decreased by 14.7 and 11.5 °C for Control and Paraffin-GGBS room models. Thus, the temperature fluctuation is reduced substantially in the Paraffin-GGBS composite room model compared to control. Based on the test results, it can be concluded that the prepared Paraffin-GGBS composite is a promising candidate for thermal energy-storage applications.

We would like to mention here that the compatibility between the cement matrix and the composite PCM (bond between them) plays an important role in evaluating the mechanical properties of a thermal energy storage cementitious composite. For example, the thermal performance of cement mortar prepared with n-octadecane/expanded graphite composite PCM was evaluated by Zhang *et al.* [46]. The thermal energy storage cement mortar (TESCM) improved the thermal performance while the compressive strength with 2.5% n-octadecane/expanded graphite composite PCM by mass of TESCM was found to be 10.5 MPa. Hunger *et al.* [47] also reported the compressive strength results of micro encapsulated PCM self-compacting concrete. It was reported that the compressive strength of self-compacting concrete with 3% microencapsulated PCM by mass of concrete was 35 MPa, which is acceptable for most constructional purposes. Recently, Xu and Li [48] produced thermal energy storage cementitious composite by incorporation of paraffin/diatomite composite PCM. With the incorporation of 30% paraffin/diatomite composite PCM, the compressive strength of thermal energy storage cementitious composite at 28-days was about 25.7 MPa, which according to the authors, is acceptable by building materials standards (Chinese National Standard GB 50574-2010) [49]. Since, the composite PCMs developed in this research would be utilized in a mortar/concrete system, it is suggested that the mechanical performance of the mortar/concrete prepared with CPCMs should also be investigated in future studies.

## 4. Conclusions

A combination of building materials and PCM is an efficient way to increase the thermal energy-storage capacity of building components for the purpose of direct thermal energy storage in buildings. Therefore, in this study, Kaolin and GGBS, which are widely used building materials, were used to develop CPCMs. The CPCMs were prepared by incorporation of paraffin into these building materials through vacuum impregnation. Based on the test results, the following conclusions can be drawn:

(1) Through vacuum impregnation, the maximum percentage of paraffin retained by Kaolin and GGBS was found to be 18% and 9%, respectively. ESEM micrographs showed that paraffin was well confined in the pores of Kaolin and GGBS through capillary forces and surface tension which, in turn, prevented the seepage of the melted PCM.
(2) FT-IR results showed that the interaction between the components of composite PCM are physical in nature and were also responsible for preventing the leakage of paraffin during its phase transition from solid to liquid. It can therefore be concluded that the components of the prepared CPCMs are chemically compatible with each other.
(3) From DSC analysis, it was found that the phase-change temperatures of the developed CPCMs are in the proper temperature range for human comfort. Therefore, it can be used in building applications as a thermal energy-storage material where it can moderate the fluctuations in indoor temperatures and improve the indoor thermal environment. Moreover, the prepared CPCMs have considerable energy-storage potential; therefore, they can be used to decrease cooling, heating, and air-conditioning loads in buildings.
(4) From TGA results, it was found that none of the prepared CPCMs showed signs of degradation below 150 °C, and almost no weight loss was observed, indicating that the prepared CPCMs are very stable in the working temperature region. Therefore, it can be concluded that the CPCMs have good thermal stability and can be used in thermal energy-storage applications.
(5) The chemical structure of prepared CPCMs was not affected by thermal cycling. Moreover, the changes observed in the phase-change temperature and latent heat storage of the prepared CPCMs after repeated thermal cycling were smaller. Therefore, the prepared CPCMs have good thermal reliability.
(6) From the self-designed thermal performance setup, it was found that the prepared CPCMs are effective in reducing the indoor temperature. Additionally, the temperature curves for the room model with CPCMs were right-shifted. Therefore, it can be concluded that CPCMs may be helpful in reducing the energy consumption by decreasing the indoor temperature and hence can be a potential candidate for applications in building facades.
